# Reliability of a Protocol to Elicit Peak Measures Generated by the Lower Limb for Semi-recumbent Eccentric Cycling

**DOI:** 10.3389/fspor.2021.653699

**Published:** 2021-05-05

**Authors:** Joel A. Walsh, Paul J. Stapley, Jonathan Shemmell, Darryl J. McAndrew

**Affiliations:** ^1^Neural Control of Movement Laboratory, Faculty of Science, Medicine and Health, School of Medicine, University of Wollongong, Wollongong, NSW, Australia; ^2^Neuromotor Adaptation Laboratory, Faculty of Science, Medicine and Health, School of Medicine, University of Wollongong, Wollongong, NSW, Australia

**Keywords:** semi-recumbent cycling, eccentric, power, workload, torque

## Abstract

Semi-recumbent eccentric (ECC) cycling is increasingly used in studies of exercise with healthy and clinical populations. However, workloads are generally prescribed using measures obtained during regular concentric cycling. Therefore, the purpose of the study was to quantify the reliability of measures derived from a protocol that elicited peak ECC torque produced by the lower limb in a semi-recumbent position. Experiments were carried out on a dynamometer in a seated, semi-recumbent position identical to that of a custom-built ECC cycle, a modified Monark recumbent cycle. Thirty healthy participants completed two testing sessions. Each session comprised three series of six repetitions of a peak ECC torque protocol (PETP) on an isokinetic dynamometer. Absolute and relative reliability of peak torque, power, angle of peak torque, and work (recorded for each repetition) was determined using coefficient of variation (CV) and intraclass correlation coefficient (ICC), respectively. Ratings of perceived exertion (RPE), muscle soreness, and perceived effort (PE) were recorded pre-PETP, immediately post-PETP, and 1-min post each PETP. The protocol showed absolute reliability values <15% for mean peak (CV = 10.6–12.1) torque, power (CV = 10.4–12.3), angle of peak torque (CV = 1.2–1.4), and work (CV = 9.7–12.1). Moderate to high between-test relative reliability is reported for mean and highest torque (ICC = 0.84–0.95; ICC = 0.88–0.98), power (ICC = 0.84–0.94; ICC = 0.89–0.98), and work (ICC = 0.84–0.93; ICC = 0.88–0.98), respectively. Within-session peak torque, peak power, and peak work showed high relative reliability for mean (ICC = 0.92–0.95) and highest (ICC = 0.92–0.97) values. Overall, the PETP test provides a reliable way of determining peak ECC torque specific to semi-recumbent ECC cycling that may be used to prescribe workloads for this form of exercise.

## Introduction

Eccentric (ECC) cycling is becoming increasingly common as a form of exercise in healthy and clinical populations and is the subject of an ever-growing number of fundamental and clinical research studies (Franchi and Maffiuletti, 2019). Indeed, at equivalent absolute workloads, cardiovascular stress is lower during ECC cycling compared to traditional concentric (CON) cycling (Dufour et al., 2004; Hoppeler, 2016). Furthermore, increased muscle size and strength can be achieved at significantly lower cardiovascular cost (Clos et al., 2019), making ECC cycling highly beneficial for patients with respiratory and cardiovascular complications (Rooyackers et al., 2003; Chasland et al., 2017; MacMillan et al., 2017; Ward et al., 2021). However, among healthy populations, the benefits of ECC cycling are less convincing and could be related to workload prescription (Coratella et al., 2019; Barreto et al., 2020). For example, workloads (intensity) for ECC cycling are commonly prescribed using measures obtained during CON cycling exercise (Franchi and Maffiuletti, 2019). These include maximal aerobic power output (Dufour et al., 2007), ventilatory threshold (Perrey et al., 2001), subjective ratings of perceived exertion (Laroche et al., 2013), or maximal aerobic heart rate (Rakobowchuk et al., 2018), as well as percentages of age-predicted maximal heart rates (Elmer et al., 2012).

For CON cycling training regimens, intensity is prescribed using measures obtained during activities involving muscle contractions occurring in the CON mode (Quod et al., 2010). However, no such metric exists that is specific to ECC cycling based on ECC muscle contractions. Therefore, ECC workloads are often prescribed from concentrically derived measures (Perrey et al., 2001; Dufour et al., 2007; Elmer et al., 2012; Laroche et al., 2013; Rakobowchuk et al., 2018; Franchi and Maffiuletti, 2019), despite previous studies having shown differences in neural control strategies between ECC and CON muscle contractions (Duchateau and Baudry, 2014). Specifically, differences in motor unit recruitment patterns and discharge rates contribute to the significantly greater mechanical loading capacity of ECC contractions compared to CON contractions (Duchateau and Baudry, 2014; Duchateau and Enoka, 2016). These neuromuscular differences likely contribute to the substantially greater mechanical loading during ECC cycling compared to CON cycling at fixed heart rate values (Lastayo et al., 1999; Dufour et al., 2004, 2007; Lipski et al., 2018).

As such, a strong argument can be made that prescribing ECC cycling workloads, based on non-specific concentrically derived measures, is likely to underestimate peak ECC capacity and reduce the efficacy of performing ECC cycling exercise (Coratella et al., 2019). Subsequently, there is a need to develop testing protocols specific to ECC cycling (Coratella et al., 2019; Barreto et al., 2020). Addressing the discrepancy of workload prescription could result in producing more convincing beneficial applications of ECC cycling among healthy populations by maximizing ECC-induced adaptations and improving the efficacy and application of ECC cycling exercise (Coratella et al., 2019; Barreto et al., 2020). Consequently, the prescription of ECC cycling, particularly among a heterogeneous healthy population, may be best served using a mechanical value derived from an ECC isokinetic test specific to semi-recumbent ECC cycling. Such a test would potentially allow for more accurate prescription of intensities specific to semi-recumbent ECC cycling. Therefore, this study aimed to determine the reliability of a protocol that measures peak ECC torque generated by the lower limb in a body position directly comparable to that during semi-recumbent ECC cycling. Relative and absolute measures of reliability were quantified for peak torque, peak power, angle of peak torque, and peak work recorded during the protocol.

## Materials and Methods

### Participants

Thirty (22 male, eight female) healthy participants (mean ± SD; age = 33.3 ± 11.4 years; mass = 75.1 ± 12.6 kg; height = 179.9 ± 8.8 cm; body mass index = 23.0 ± 3.0 kg·m^−2^) with no history of neurological, orthopedic, or cognitive impairment volunteered to participate in this study. Our sample size was comparable to or greater than studies investigating the reliability of peak ECC knee extensor torque (Maffiuletti et al., 2007) and power output during ECC cycling (Brughelli and Van Leemputte, 2013). All participants were right leg dominant and were moderately physically active (mean score: 11,506 METS) according to the International Physical Activity Questionnaire (Booth, 2000). The University of Wollongong Human Research Ethics Committee (ethics number: 2018/347) approved all experiments that were carried out in accordance with the Declaration of Helsinki (World Medical Association, 2013).

### Design

**Figure 1:**
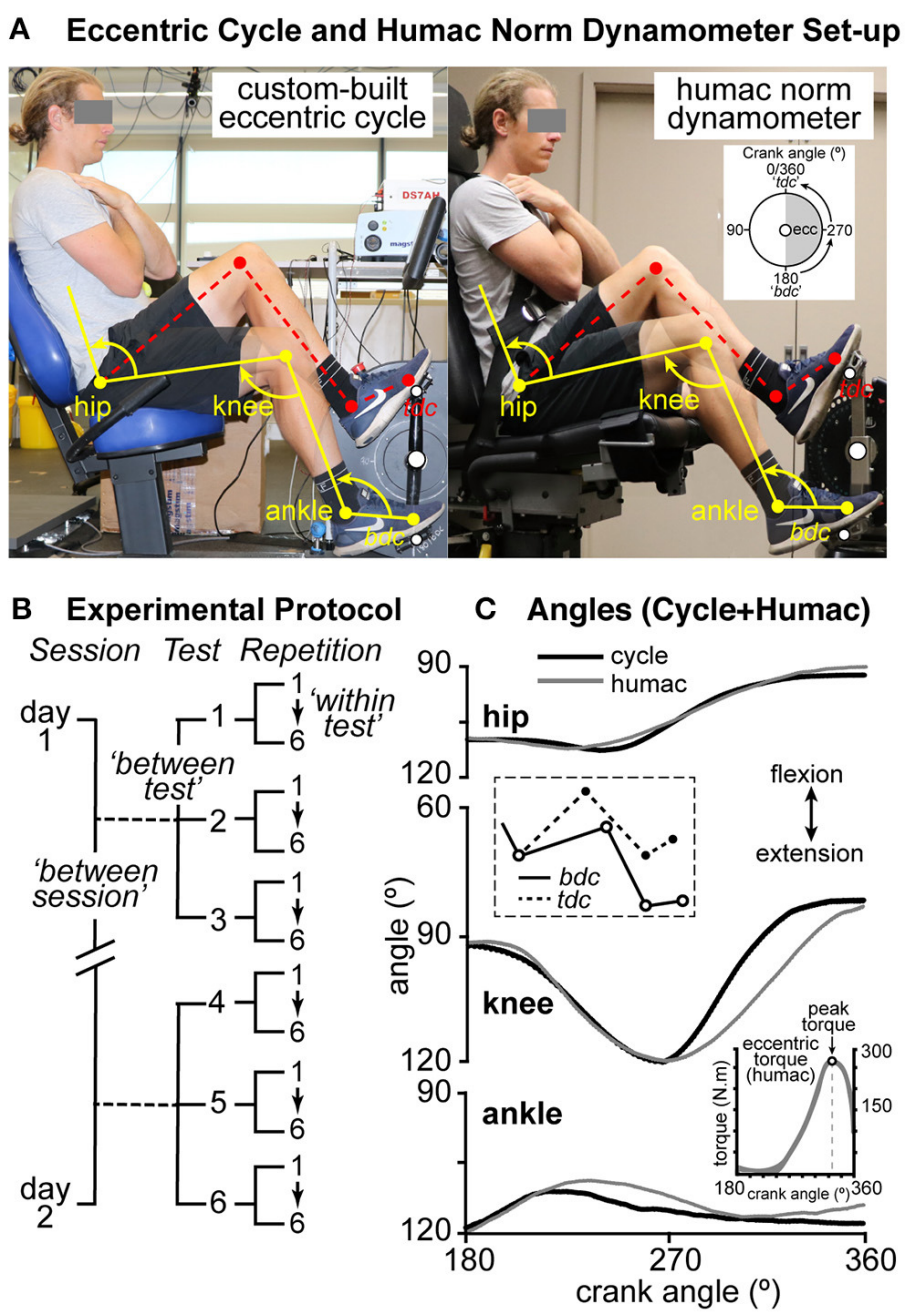
**(A)** A subject seated on the custom-built eccentric (ECC) cycle (left) and isokinetic dynamometer (right). Hip, knee, and ankle angles are illustrated at bottom dead center (“bdc,” yellow) and top dead center (“tdc,” red dashed lines). Inset (right side) illustrates the approximate area in which ECC torque was exerted on the dynamometer (shaded area). **(B)** Outline of the experimental protocol (see *Materials and Methods*). **(C)** Mean hip, knee, and ankle angles for one subject between 180° (bdc) and 0/360° (tdc) for both the semi-recumbent cycle (black lines) and Humac dynamometer (gray lines). Inset (bottom right) represents the mean torque recorded by the isokinetic dynamometer for one peak ECC torque protocol (PETP) (six repetitions).

This study used a repeated measures experimental design to quantify the test–retest reliability of a novel peak ECC torque protocol (PETP) test. A six-repetition protocol (Figure 1B) was used to assess muscle strength, as it is highly reliable and predictive of one repetition strength (Reynolds et al., 2006). Participants performed three voluntary PETP tests on two separate testing days, completing a total of six individual tests over 2 days (i.e., two sessions, three tests per session, six repetitions per test, totaling 36 repetitions; Figure 1B). The two experimental sessions were separated by 48 h, and the sessions occurred at the same time on both days. Participants refrained from ingesting caffeine or alcohol and intense physical activity 12 and 24 h, respectively, prior to their tests. Testing was carried out under standard laboratory conditions (20–22°C; ~50% relative humidity) (Pina et al., 1995).

Each session began with participants completing a warm-up consisting of 10 min of CON semi-recumbent cycling at a low intensity (between 40 and 50% of age-predicted maximal heart rate) at 60 rpm. Participants were then seated on the dynamometer, and a Velcro strap was fastened around their chest to minimize upper body movement. Each subject performed four tests to familiarize themselves with the movement. In the first two of the familiarization tests, the subject provided no resistance to the pedal movement (passive experience of the test) and, during the second test, resisted to ~50% of their self-predicted maximal effort. After 1 min of rest, three PETP tests (one test = six repetitions) were completed, with each test separated by 2.5 min rest. Participants were instructed to cross their arms over their chest while performing the test and maximally resist against the counterclockwise (CCW) movement of the pedal arm using the visual feedback of torque and verbal encouragement from the researchers to improve subsequent repetitions if possible.

### Equipment

The experimental setup is shown in Figure 1A. An isokinetic dynamometer (Humac, Computer Sports Medicine Inc., Stoughton, MA, USA) measured the combined torque exerted around the hip, knee, and ankle joints (see Figure 1A, right side). The dynamometer reproduced a seat angle of 100°, a seat width of 300 mm at its widest point, and a bottom bracket drop of 200 mm, which corresponded to the dimensions of a Monark AB837E semi-recumbent cycle ergometer (Monark Exercise AB, Vansbro, Sweden; Figure 1A, left side) that has been modified as an ECC cycle ergometer (*manuscript in preparation*). Briefly, the Monark semi-recumbent cycle was fitted with two direct drive servo motors (Hans type FI3-015-S-A-1, Motion Technologies, Caringbah, NSW, Australia) that could drive the pedals in a CCW direction.

The dynamometer was programmed to move through a 180° range of motion in a CCW direction (see shaded area of inset, Figure 1A, right side) at an angular velocity (ω) of 6.283 rad·s^−1^ (60 rpm; 360°·s^−1^ velocity) and reset in its initial position every 6 s. The dynamometer recorded numerical data for torque, position, and time. Power (watts) data were calculated by the dynamometer using the following equation (Computer Sports Medicine, Inc., HUMAC2009®/Norm™, Application Program–User's Guide):

(1) $$\begin{array}{l} Power\;\left(watts\right)=\frac{\text{Work }\left(\text{ft}-\text{lbs}\right)}{\text{Time }\left(\text{s}\right)}\times 1.3558179 \end{array}$$

Torque data, corrected for gravity, were collected continuously throughout each test. The 180–360° range of motion was used to assess peak ECC torque, as peak torque during semi-recumbent ECC cycling has been reported to occur at ~300–325° (Green et al., 2018). The inset of Figure 1C shows the mean occurrence of peak ECC torque in the representative participant.

Seat position was identical between the ECC cycle and dynamometer when the foot was at its farthest extended point (not shown in Figure 1). For both the cycle and dynamometer, in this position, the hip angle was 105–110° and the knee angle was 130–170°, which corresponded to the configuration of Elmer et al. (2010). Hip, knee, and ankle angles at the “bottom dead center” (the foot at the bottom of the crank cycle, or *bdc*) and at the “top dead center” (the foot at the top of the crank cycle, or *tdc*) for the ECC cycle and the dynamometer are shown in Figure 1A (left and right panels, respectively). Mean joint angles (from six ECC pedal movements and six trials on the dynamometer) for one representative participant at the hip, knee, and ankle are shown in Figure 1C. Angles were very similar between the dynamometer and the semi-recumbent ECC cycle.

### Data Recording

Performance variables including torque (N·m), power (Watts, W), work (joules, J), and angle of peak torque (°) were recorded for each test and exported for offline analysis. For clarity, a representative trace of dynamometer torque as a function of crank angle is shown in Figure 1C. Muscle soreness and perceived effort (PE) were explained to participants as the level of pain within the quadriceps and “*the amount of mental or physical energy being given to a task*” (Penailillo et al., 2017) and measured pre-, immediately post, and 1 min post each test using a 100 mm visual analog (VAS) scale (0 represented no soreness/effort, and 10 represented maximal soreness/effort). Perceived exertion was explained to participants as the “*degree of heaviness and strain experienced during physical work*” (Penailillo et al., 2017) and recorded immediately after and 1 min after each test using the Borg's Rating of Perceived Exertion (RPE) 6–20 scale (Borg, 1998).

### Data Analysis

A 2 × 6 repeated measures ANOVA [2 days/session and six tests (each six repetitions)] was used to test for differences in peak torque, power, angle of peak torque, and work between tests and within sessions. Absolute reliability and relative reliability of peak performance variables (torque, power, angle of peak torque, and work) were assessed using coefficient of variation (CV) and intraclass correlation coefficient (ICC), respectively. Intrasubject, within-test absolute reliability (CV) was calculated as the standard deviation (SD) of the six repetitions (within tests) divided by the mean of those six repetitions. Intrasubject, within-session absolute reliability (also CV) was calculated as the SD of all 18 repetitions (within each session/day) divided by the means of all 18 repetitions. Each was expressed as a percentage.

ICC values were used to determine intrasubject between-test, within-session, and between-session relative reliability of performance measures. Values <0.80, between 0.80 and 0.90, and >0.90 were considered to have questionable, moderate, and high reliability (Maffiuletti et al., 2007), respectively. ICCs were performed on the highest and mean values of each set of six repetitions. Minimal detectable change (MDC) values were calculated using the following equation:

(2) $$\begin{array}{l} MDC=SEM\;\times 1.96\times \;\sqrt{2} \end{array}$$

According to this equation, the standard error of measurement (SEM) was calculated as SD × √(1-ICC), and 1.96 is the z-score for the 95% confidence interval (CI) (Ries et al., 2009). MDC values were used as an indication of the minimal amount change in values that represent meaningful change (Ries et al., 2009). Data are presented as mean ± SD or ranges and presented as CV and ICC with 95% CIs in tables and figures. SPSS Statistics for Windows (IBM, Version 23.0, IBM Corp., Armonk, NY, USA) was used for all ANOVA and ICC statistical analyses. Microsoft Excel 2016 (Microsoft Corp., Redmond, WA) was used to calculate SEM and MDC values. Statistical significance was set at *p* < 0.05.

## Results

### Ratings of Perceived Exertion, Muscle Soreness, and Perceived Effort

Mean measures (all participants) of RPE, muscle soreness, and PE collected immediately post each of the six tests ranged from 9.1 to 10, 1.4 to 1.8, and 1.9 to 2.8, respectively. Mean values across each of the six tests for all participants were 9.5 ± 2.5, 1.5 ± 1.3, and 2.3 ± 1.8 (RPE, muscle soreness, and PE, respectively).

### Torque, Work, and Power Within and Between Tests

**Figure 2:**
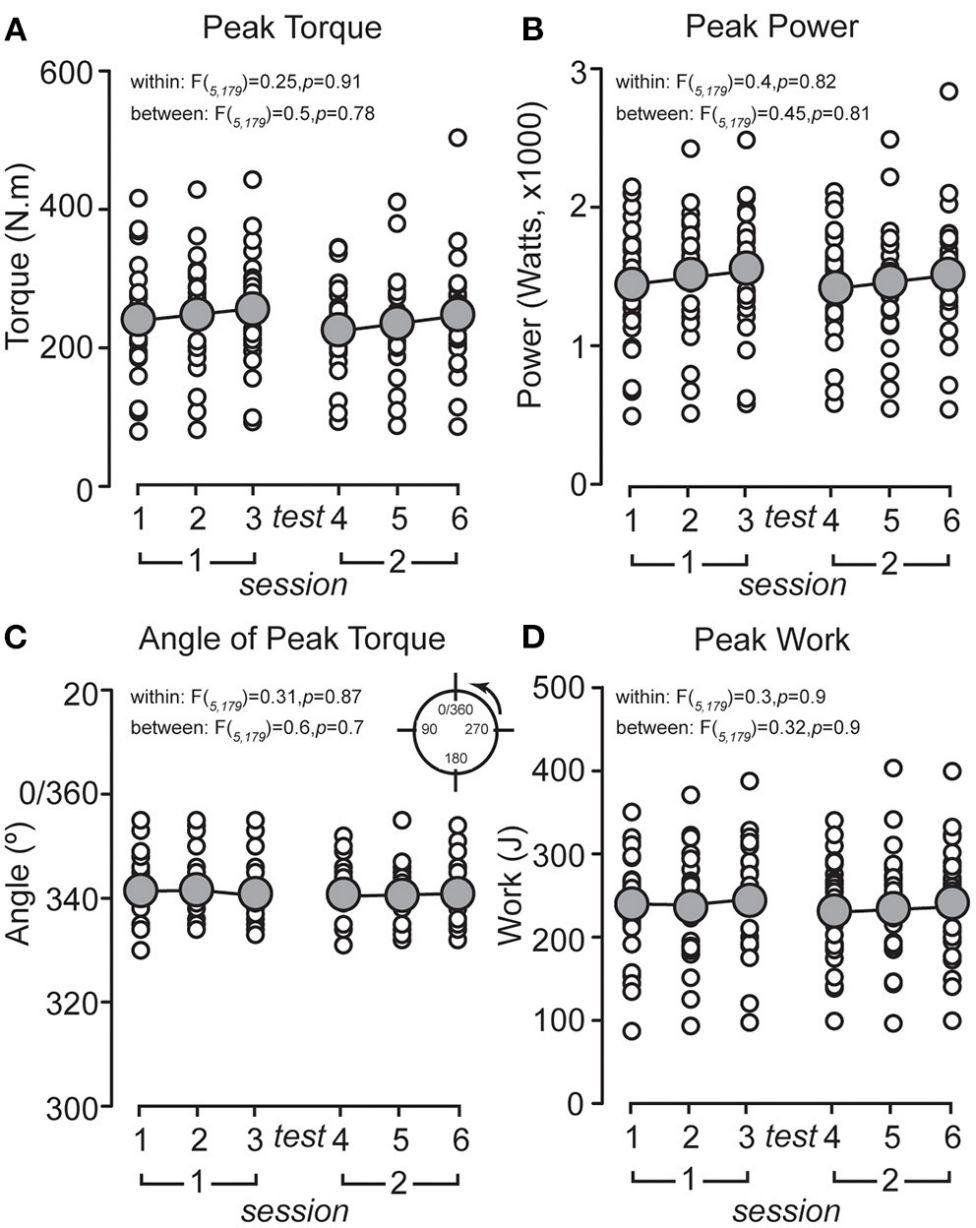
Mean peak (of six repetitions) data for all participants (*n* = 30) plotted for each peak eccentric torque protocol (PETP) for **(A)** peak torque, **(B)** peak power, **(C)** angle of peak torque, and **(D)** peak work. The population mean for each test is represented by the gray circle.

Mean and individual max torque, power, angle of max torque, and work data recorded during each PETP for all participants are shown in Figures 2A–D, respectively. Across all six tests, peak torque ranged from 263.4 ± 71.4 N·m to 292.7 ± 81.7 N·m (mean of the six tests: 278.8 ± 82.2 N·m), the angle of peak torque from 340.5° ±5.6° to 342.3° ±6.1° (mean: 341.2° ± 5.6°), peak power from 1,617.7 ± 415.8 W to 1,777.5 ± 443 W (mean: 1,690.5 ± 448.4 W), and peak work from 255.3 ± 62.0 J to 276.2 ± 64 J (mean: 268.5 ± 67.3 J). ANOVA revealed that there were no within-test or between-session differences for any of the variables (F and *p*-values are shown in Figure 2).

### Absolute Reliability of Torque, Work, and Power

**Figure 3:**
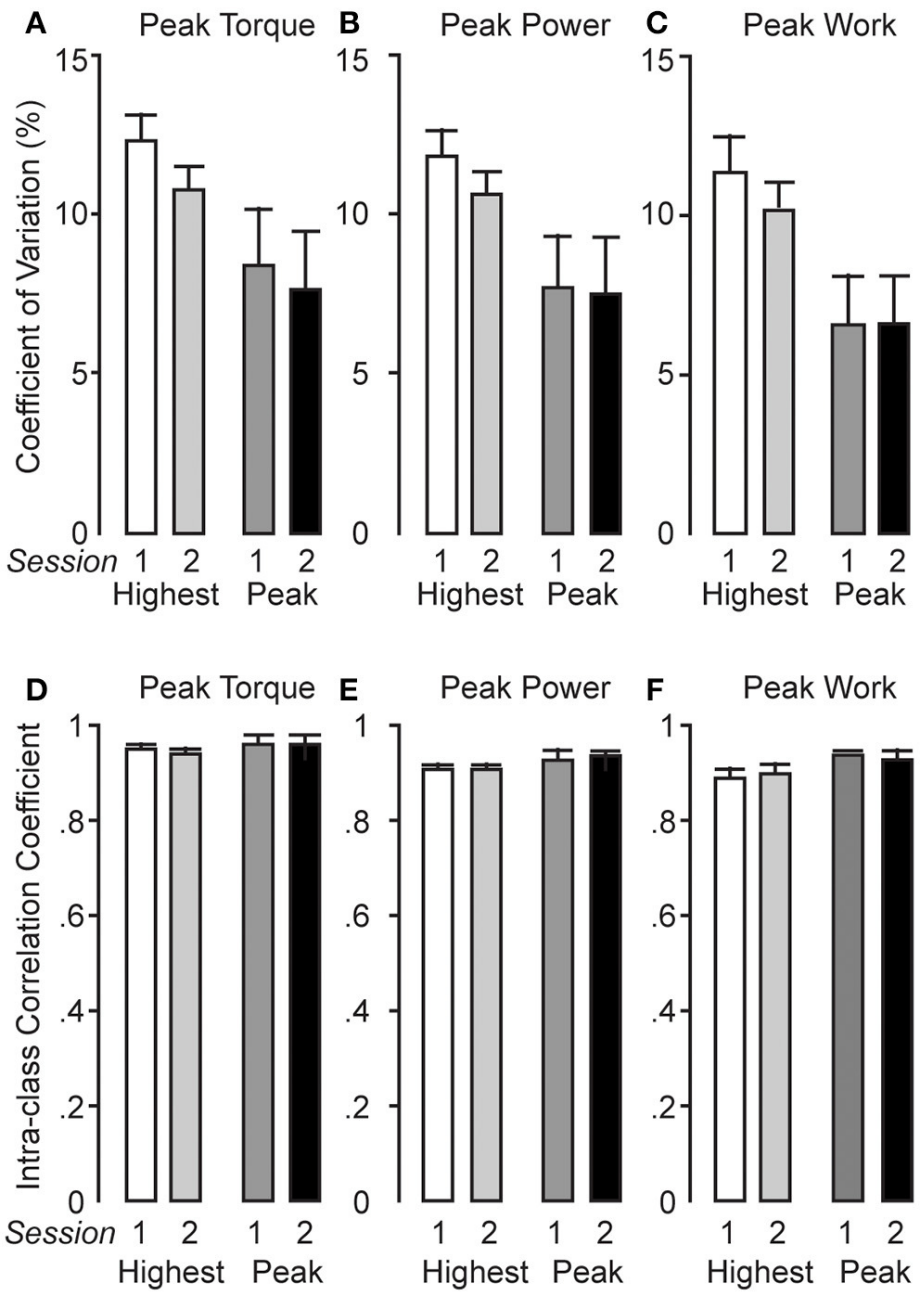
Between-session coefficient of variation (CV) ± 95% CI **(A–C)** and intraclass correlation coefficient (ICC) ± 95% CI (**D–F**) values are presented for mean and highest torque, power, and work [i.e., session 1 represents peak eccentric torque protocols (PETPs) 1–3, and session 2 represents PETPs 4–6].

Mean CV values for within-test peak torque, power, angle of peak torque, and work are shown in Table 1. Coefficient of variation values for mean within-test reliability showed levels of absolute reliability of <15%. Mean peak torque CV values ranged from 10.6 to 12.7, power from 10.4 to 12.3, angle of peak torque from 1.2 to 1.4, and work from 9.7 to 12.1. In fact, the CV values for mean within-test peak torque, power, and work generally increased their absolute reliability by test 5. Mean CV values for the angle of peak torque showed very high absolute reliability (<2%) from tests 1 to 6. Between-session mean and peak CV values for all performance variables showed acceptable (<15%) to high (<10%) absolute reliability (Figures 3A–C).

**Table 1:** Mean CV (%) values with their respective lower and upper limits (95% confidence interval) representing within-test absolute reliability of the PETP. Subscript number denotes the respective PET test.

	**Peak torque (N·m)**	**Peak power (W)**	**Angle of peak torque (°)**	**Peak work (J)**
**Mean within-test absolute reliability**				
CV_1_	12.7 (11.1–14.2)	12.3 (10.7–13.8)	1.4 (1.4–1.6)	11.0 (9.2–12.8)
CV_2_	12.6 (11.1–14.0)	11.8 (10.3–13.3)	1.3 (1.1–1.5)	12.1 (9.8–14.4)
CV_3_	12.0 (10.9–13.2)	11.7 (10.5–12.8)	1.2 (1.0–1.4)	11.7 (10.3–13.1)
CV_4_	11.1 (9.7–12.4)	11.0 (9.7–12.4)	1.2 (1.1–1.4)	11.0 (9.2–12.7)
CV_5_	10.6 (9.6–11.6)	10.4 (9.2–11.6)	1.2 (1.1–1.3)	9.7 (8.4–10.9)
CV_6_	11.1 (9.7–12.4)	10.7 (9.3–12.1)	1.2 (1.0–1.3)	10.4 (9.2–11.7)
Mean CV	11.7 (10.4–13.0)	11.3 (9.9–12.7)	1.3 (1.1–1.4)	11.0 (9.4–12.6)

*Subscript number denotes the respective PETP test*.

*CV, coefficient of variation*.

### Relative Reliability of Torque, Work, and Power

ICC values for mean and peak between-test torque, power, angle of peak torque, and work are presented in Table 2. Between-test ICC values for *mean* torque (MDC = 72.1 ± 52.0–95.6 N·m), power (MDC = 404.5 ± 301.5–509.5 W), and work (MDC = 64.4 ± 53.0–77.1 J) showed moderate (0.80–0.90) to high (>0.90) test–retest reliability. Moreover, between-test ICC values for *highest* peak torque (MDC = 57.3 ± 32.9–82.8 N·m), peak power (MDC = 300.8 ± 169.8–402.2 W), and peak work (MDC = 37.2 ± 35.1–40.1 J) values also showed moderate–high (ICC >0.90) test–retest reliability in most cases from the first to the sixth test. Within-session mean and highest ICC values for peak torque, peak power, and peak work (Figures 3A–C) showed high reliability ranging from 0.92 to 0.95 for mean values and between 0.92 and 0.97 for highest values. Between-session ICC values for mean and highest torque, power, and work showed moderate (range = 0.84–0.87) to high reliability (range = 0.90–0.92; Figures 3D–F), respectively. From Table 2, it can be seen that, generally, the angle of highest (MDC = 8.8° ± 6.9°-10.1°) and mean (MDC = 7.9° ± 6.5°-10.7°) angles of peak torque had questionable reliability (on average 0.66 and 0.73, respectively).

**Table 2:** Between-test relative reliability (ICC values with lower and upper limit 95% CI) for highest and mean peak torque (N·m), peak power (W), angle of peak torque (°), and peak work (J).

	**Peak torque (N·m)**		**Peak power (W)**		**Angle of peak torque (**^****°****^**)**		**Peak work (J)**	
	**Highest**	**Mean**	**Highest**	**Mean**	**Highest**	**Mean**	**Highest**	**Mean**
**Peak and mean between-test relative reliability**								
ICC_1−2_	0.92 (0.84–0.96)	0.90 (0.86–0.93)	0.93 (0.86–0.97)	0.89 (0.85–0.92)	0.78 (0.54–0.89)	0.76 (0.68–0.82)	0.96 (0.91–0.98)	0.87 (0.82–0.91)
ICC_2−3_	0.98 (0.95–0.99)	0.95 (0.93–0.96)	0.98 (0.94–0.99)	0.94 (0.92–0.96)	0.83 (0.64–0.92)	0.84 (0.79–0.88)	0.98 (0.95–0.99)	0.91 (0.88–0.93)
ICC_3−4_	0.89 (0.66–0.96)	0.86 (0.72–0.91)	0.90 (0.68–0.96)	0.85 (0.74–0.91)	0.62 (0.21–0.82)	0.74 (0.65–0.81)	0.88 (0.70–0.95)	0.84 (0.76–0.89)
ICC_4−5_	0.95 (0.89–0.97)	0.91 (0.88–0.93)	0.94 (0.88–0.97)	0.91 (0.87–0.93)	0.53 (0.01–0.78)	0.71 (0.61–0.78)	0.92 (0.83–0.96)	0.88 (0.83–0.91)
ICC_5−6_	0.96 (0.92–0.98)	0.92 (0.88–0.94)	0.96 (0.93–0.98)	0.92 (0.88–0.94)	0.61 (0.18–0.81)	0.80 (0.74–0.85)	0.97 (0.95–0.99)	0.93 (0.90–0.95)
ICC_1−6_	0.88 (0.75–0.94)	0.84 (0.78–0.88)	0.89 (0.77–0.95)	0.84 (0.78–0.88)	0.61 (0.21–0.81)	0.56 (0.41–0.67)	0.90 (0.79–0.95)	0.84 (0.79–0.89)
Mean ICC	0.93 (0.84–0.97)	0.89 (0.84–0.93)	0.93 (0.84–0.97)	0.89 (0.84–0.92)	0.66 (0.30–0.84)	0.73 (0.70–0.80)	0.93 (0.84–0.97)	0.88 (0.83–0.91)

*ICC_1−2_ denotes test (one vs. test two) and ICC_1−6_ denotes first vs. last (i.e., one vs. six)*.

*ICC, intraclass correlation coefficient*.

## Discussion

This study presents an isokinetic test developed to determine peak ECC torque through a range of motions directly comparable to that experienced during semi-recumbent ECC cycling. The findings show that the protocol is a reliable way of determining peak isokinetic ECC torque and, hence, power. High reliability was achieved with minimal perceived exertion, muscle soreness, and perceived effort. Overall, the PETP test is a reliable and straightforward protocol with which to determine peak ECC torque obtained under conditions that exactly replicate a semi-recumbent ECC cycling position and from which ECC-specific workloads can be prescribed.

### Comparison of Peak Eccentric Torque Test With Other Eccentric Strength Tests

Our results show similar levels of reliability of extensor torque recorded on an isokinetic dynamometer during a traditional maximal isokinetic ECC strength test (Maffiuletti et al., 2007). These authors reported moderate to high relative reliability (ICC = 0.97–0.99) for peak torque and power during maximal isokinetic ECC contractions. Despite differences in movement velocity (60°·s^−1^ in their study vs. 360°·s^−1^ in the present study) and number of repetitions (three in Maffiuletti et al., 2007 vs. six in ours), the findings of both studies show comparable levels of reliability for peak torque, power, and work. This would suggest that the PETP produces comparable outcomes to a traditional isokinetic ECC strength test of the knee extensors in an experimental setup that is adapted to the actual semi-recumbent ECC cycling position. However, it is important to note that differences in ECC cycling movement velocity impacts force production and muscle damage (Ueda et al., 2020). In their study, participants performed 5 min of ECC cycling at fast (210°·s^−1^) and slow (30°·s^−1^) velocities, with fast-velocity ECC cycling significantly impairing muscle strength and increasing muscle soreness compared to slow-velocity ECC cycling. In contrast, the PETP test, performed at a velocity of 360°·s^−1^ (ω = 6.283 rad·s^−1^, equivalent to cycling at 60 rpm), resulted in minimal muscle soreness and did not adversely affect the reliability of peak torque and power measures and, therefore, muscle strength. Muscle strength and soreness difference between the PETP test and fast-velocity ECC cycling could be due to the duration of the tasks (i.e., 6 s for the PETP test vs. 5 min or 300 s^−1^ for fast-velocity ECC cycling) and the subsequent differences in their cumulative loads. It is not unexpected that the 6-s duration PETP test does not negatively affect muscle strength or induce substantial muscle soreness given that ECC exercise is often performed in low-repetition sets (Suchomel et al., 2019b) to likely minimize the impact of exercise-induced muscle damage (Suchomel et al., 2019a). Additionally, high within-test absolute reliability for raw angle of peak torque (mean CV = 1.3; 1.1–1.4%) data (Figure 2C) obtained across the six PETP tests in our study would suggest minimal change in the population average, given that the mean range of angle of peak torque (340–342°) fell within the ECC pedaling phase (see *Materials and Methods* and Figure 1). Furthermore, averaged MDC values reported for between-test highest and mean peak torque, power, and work could be interpreted as the minimum resistance (i.e., torque, power) and work required to signify real change within a group (Furlan and Sterr, 2018), performing the PETP test. However, given that there is no published criteria for interpretation, care should be exercised when analyzing MDC values (Ries et al., 2009; Wilken et al., 2012; Dontje et al., 2018) reported for the PETP test.

In the current study, participants performed six repetitions during a PETP at an angular velocity of 360°·s^−1^ that equated to an approximate total test time of 6 s, analogous to the 6-s maximal sprint cycling test (Mendez-Villanueva et al., 2007). The reliability of the six-repetition PETP is in agreement with the findings of the aforementioned studies (see *Materials and Methods*) that used six-repetition and 6-s testing protocols (Brughelli and Van Leemputte, 2013; Chasland et al., 2017). Indeed, Chasland et al. (2017) showed acceptable levels of variation in peak (CV = 8–9%) and average (CV = 4–5%) power output when performing maximal semi-recumbent ECC cycling at 60 rpm, which are comparable to the variances reported in the current study for mean within-test power (mean CV = 11.3; 9.9–12.7%). Additionally, Brughelli and Van Leemputte (2013) assessed the reliability of power output during upright ECC sprint cycling, reporting moderate to high levels of reliability for mean (ICC = 0.83) and peak (ICC = 0.96) power outputs after two familiarization tests, similar to our findings (Tables 1, 2). These authors suggested that a learning effect was responsible for the improved reliability during consecutive ECC sprint tests. Given the novelty of our PETP, it is also likely that a learning effect occurred that may explain the reduced absolute reliability of mean torque (CV = 12.7 to 10.6%), power (12.3 to 10.4%), and work (11 to 9.7%) between tests 1 and 5 (Table 1). Despite any small differences, the highly acceptable levels of absolute and relative reliability for torque, power, and work were evident from the first PETP. Of note, however, was the lower reliability of the highest and mean values of the angle of peak torque. This may have been due to no specific instruction being given to exert a peak torque at a particular time during the backward movement, rather only to produce peak force against it. Nevertheless, angular values showed a tight range (~2°) and occurred within the ECC phase.

### Situating Previously Reported Workloads Relative to Peak Eccentric Torque Test Values

However, while the benefits of submaximal ECC cycling exercise for clinical populations are well-understood, any potential benefits among healthy and athletic populations are as yet unconvincing (Paulsen et al., 2019). It has been recently suggested that prescribing semi-recumbent ECC cycling intensity based on CON-derived measures likely results in an underestimation of workload and potentially limits the efficacy of ECC-induced adaptations in healthy populations (Coratella et al., 2019). From measures of mean peak power calculated from the entire cohort of the present study, we quantified the wattages adopted by a total of 21 studies (see Supplementary Material Online for details). The reviewed prescribed workloads of studies based on the following criteria: (1) healthy participants (under 50 years) free of impairment or clinical condition, (2) adoption of a semi-recumbent ECC training or single-visit protocol, and (3) prescription of ECC workload (quantified in Watts) based on RPE, maximal aerobic power output (derived from incremental step test), maximal cycling power, age-predicted maximal heart rate, peak oxygen consumption, or peak heart rate were all obtained in CON conditions.

**Figure 4:**
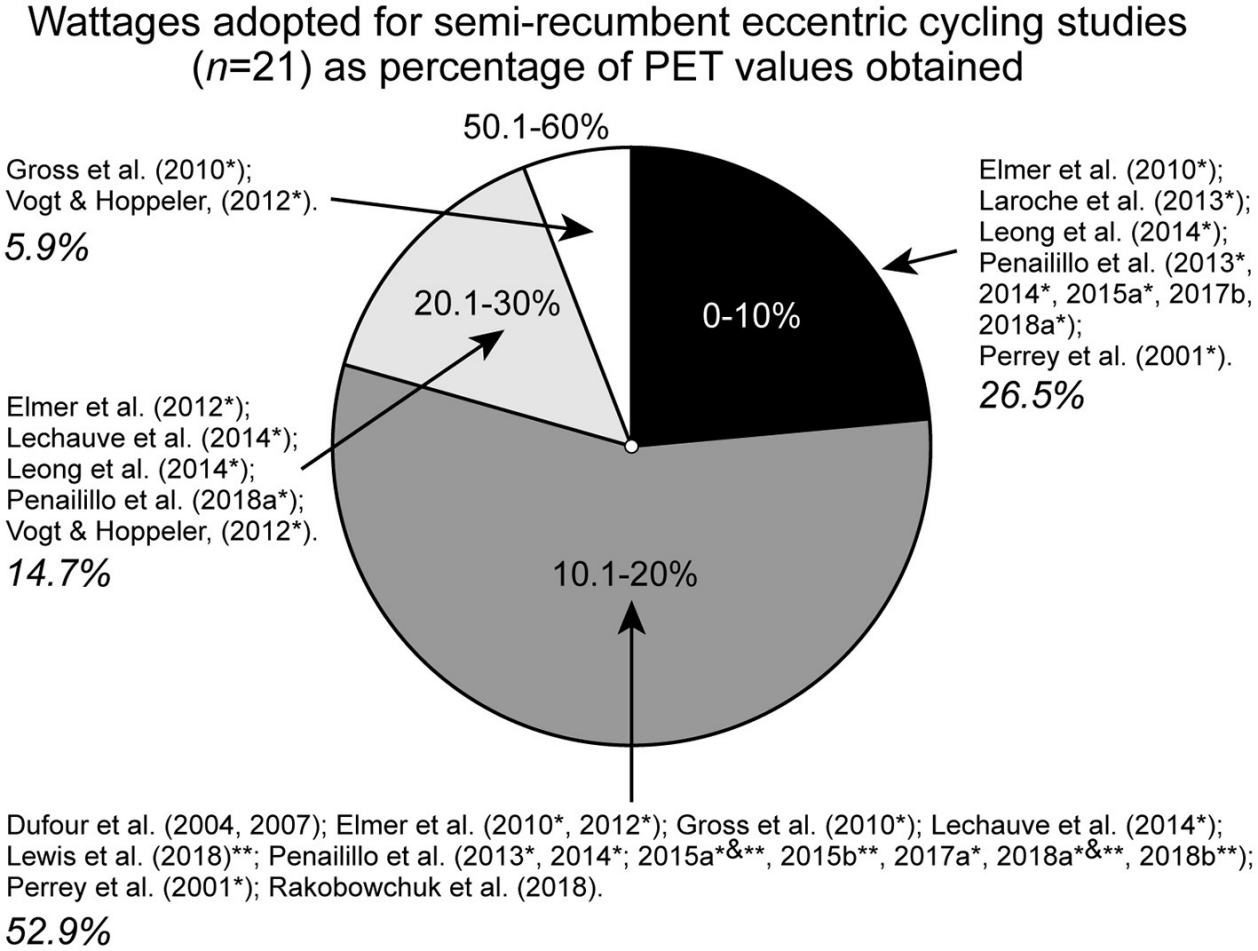
A relative comparison of the group mean for peak eccentric (ECC) torque protocol (PETP) test power (1,691 ± 448 W) with previous studies (*n* = 21) that have used non-ECC measures to prescribe semi-recumbent ECC cycling intensities (i.e., power outputs as wattages; *n* = 34). Overall, 23.5% of studies were between 0 and 10% PETP power, 55.9% between 10.1 and 20% PETP power, 14.7% between 20.1 and 30% PETP power, and 5.9% between 50.1 and 60% PETP power. *denotes that the study has multiple reported power output values that fall into *different* PETP test percentage categories. **denotes that the study has multiple reported power output values that fall into the *same* PETP percentage category.

Figure 4 quantifies the workloads in watts (including any first or final session or beginning/end test values, hence, *n* = 34) from the 21 studies (different group workloads in some studies) expressed as a percentage of mean PETP power output (mean = 1,690.5 ± 448.4 W) obtained in the present study. A total of 94.1% of studies prescribed semi-recumbent ECC cycling workloads at <30% of the PETP power obtained in the present study. As such, if participants from our study were prescribed ECC cycling workloads utilized in the reviewed studies, they would be cycling at workloads <30% of the mean PETP power and, in some cases, <10% that could be interpreted as low intensity. Studies that used lower intensities (<10%) relative to the PETP test often reported mixed results, ranging from reduced maximal power output (Elmer et al., 2010) and decreased or no change in maximal voluntary contraction and countermovement jump (CMJ) and muscle soreness (Peñailillo et al., 2013, 2015a,b). Two studies (5.9% of the 21 studies) reported ECC cycling power output >30 % PETP power output (the 50.1–60% of mean PETP power output). These training studies reported increases in quadriceps hypertrophy, CMJ, and squat jump height and power (Gross et al., 2010; Vogt and Hoppeler, 2012). Comparatively, these findings would suggest that training adaptations among healthy populations are greater for studies that used higher ECC cycling workloads relative to the PETP test (i.e., >50% PETP). Muscle strength and size adaptations in these studies are not unexpected, as ECC resistance training protocols typically adopt high-load/low-repetition protocols in order to improve neuromuscular adaptations and muscle strength gains (Suchomel et al., 2019a,b). However, it is important to consider the differences in experimental protocols used in the 21 studies, namely, training vs. single-visit studies. Nonetheless, given the specific physiological, neuromuscular, and muscle force contractile differences between ECC and CON cycling outlined previously (Duchateau and Baudry, 2014; Clos et al., 2019; Suchomel et al., 2019a), prescribing ECC cycling intensities based on CON measures may not be task-specific and may limit possible conclusions of studies and effectiveness of interventions.

## Conclusions

In conclusion, this study aimed to develop a testing protocol that reliably measures peak isokinetic ECC torque and power specific to semi-recumbent ECC cycling. Our findings suggest that acceptable absolute and relative reliability can be achieved within one session with as little as six repetitions or less. This indicates that the PETP requires minimal learning and can therefore be easily applied by researchers and practitioners who use semi-recumbent ECC cycling in clinical or laboratory settings and have access to an easily modifiable isokinetic dynamometer. Taking into account the well-documented differences when comparing CON and ECC exercise, including cycling (Herzog, 2014; Clos et al., 2019), previous studies using CON methods to determine ECC cycling workloads may have underestimated specific intensities needed to induce training adaptations that would translate into improved performance outcomes, particularly in healthy populations (Paulsen et al., 2019). Future studies should investigate the validity of using the PETP test to prescribe semi-recumbent ECC cycling workloads, ranging from low to high intensities. If valid, the PETP test could be used to prescribe more specific and less variable semi-recumbent ECC cycling intensities at comparatively lower levels of perceived exertion, effort, and muscle soreness.

### Practical Applications

The PETP test could be used to more practically and accurately prescribe fixed ECC cycling workloads based on peak torque (N·m) and/or power output (W) values recorded by the dynamometer. Given that semi-recumbent ECC cycling ergometers typically display and record power output (W), the translational mechanical measures of the PETP test are high. This would likely minimize ambiguity of prescribing workloads using other measures of intensity (Barreto et al., 2020). As such, the PETP test would better enable researchers, coaches, and sports practitioners to plan, periodize, track, and measure semi-recumbent ECC cycling training and performance outcomes. Improving the application of semi-recumbent ECC cycling, by more specifically prescribing workloads, could better improve strength and power adaptations and, therefore, functional performance among healthy and athletic populations.

## Data Availability Statement

The raw data supporting the conclusions of this article will be made available by the authors, without undue reservation.

## Ethics Statement

The studies involving human participants were reviewed and approved by University of Wollongong, Human Research Ethics Committee. The patients/participants provided their written informed consent to participate in this study.

## Author Contributions

All the authors contributed to writing the manuscript, suggesting improvements to manuscript, reviewing the manuscript, and approved the final version of the manuscript.

## Conflict of Interest

The authors declare that the research was conducted in the absence of any commercial or financial relationships that could be construed as a potential conflict of interest.
