# Supplementary material for: Reliability of a Protocol to Elicit Peak Measures Generated by the Lower Limb for Semi-recumbent Eccentric Cycling
Source: Front Sports Act Living. 2021 May 5;3:653699. doi: 10.3389/fspor.2021.653699 (PMC8132294; doi:10.3389/fspor.2021.653699)
Supplement: Supplementary file 1 [file Table_1.DOCX]

***Supplementary Material***

The reviewed 21 studies training or single-visit studies that report ECC semi-recumbent cycling power output values (*n* = 34) prescribed using non-ECC measures. The first five studies (i.e. Gross et al., 2010 to Lewis et al., 2018) are training studies. Citations (Scopus index) are current as of 12/2019.

| Study, year | Population | Protocol | Method of prescribing ECC cycling intensity | Prescribed power (W, mean ± SD) | Number of ECC power (order number) values reported in study | Power relative (%) to mean PET test *(1691 ± 448 W)* | Study outcomes | Citations (Scopus index) |
| --- | --- | --- | --- | --- | --- | --- | --- | --- |
| Gross et al. ^1^, 2010  *“Effects of eccentric cycle ergometry…”* | *n=*15 junior male skiers, 18 ± 1 years, 74 ± 8 kg, allocated to ECC (8) or control (7) groups | 6-weeks (duration), 3×/week, 20 min intervals, 60-80 rpm | Constant or variable ECC cycling workload, progressed to a perceived exertion of “hard” | First session = 213 ± 23 W  Final session = 850 ± 71 W | 2 (1,2) | First session = 12.6 ± 1.4 %  Final session = 50.3 ± 4.2 % | ↑ quadriceps hypertrophy  ↑ CMJ and SJ  No Δ ISO leg strength | 30 |
| Vogt and Hoppeler ^2^, 2012  *“Competitive alpine skiing: combining…”* | *n=*5 world-class alpine skiers | 5-weeks, 1-2×/week, 5 min intervals | None reported | First session = 404 W  Final session = 965 W | 2 (3,4) | First session = 23.9 %  Final session =57.1 % | ↑ CMJ and SJ power  ↑ CMJ and SJ  ↑ ISO leg strength | 2 |
| Elmer et al. ^3^, 2012  *“Improvements in multi-joint leg…”* | *n*=12, 25 ± 6 years, 77 ± 23 kg, allocated to ECC (6) or CON (6) groups | 7-weeks, 3×/week, 10-30 min intervals, 60 rpm | Percentage of age-predicted maximal heart rate (MHR) | 54-77% MHR  Power progressed from 210 ± 25 W to 408 ± 53 W | 2 (5,6) | 12.4 ±1.5 % and 24.1 ± 3.1 % | ↑ CMJ P_max_  ↑ leg stiffness  No Δ P_max_ | 23 |
| Leong et al. ^4^, 2014  *“Chronic eccentric cycling improves…”* | *n*=8, 22 ± 2 years, 69 ± 13 kg | 8-weeks, 2×/week, 5-10.5 min intervals, 60 rpm | Maximal CON cycling power output (P_max_) | 20-55% of P_max ;_ Power progressed from 157 ± 24 W to 442 ± 56 W | 2 (7,8) | 9.3 ± 1.4 and 26.1 ± 3.3 % | ↑ quadriceps hypertrophy/thickness  ↑ P_max_ | 12 |
| Lewis, Peoples, Groeller & Brown ^5^, 2018  *“Eccentric cycling emphasising a low…”* | *n* =17 sedentary males allocated to ECC (9) or CON (8) cycle training | 8-weeks, 2×/week, 10-30 min ECC cycling. | Incremental step-test on semi-recumbent ergometer (in CON mode). ECC cycling at 60% P_max_ | ECC  Pre 301 ± 15 W  Post 303 ± 15 W | 2 (9,10) | ECC  Pre 17.8 ± 0.9 %  Post 17.9 ± 0.9 % | No Δ VO_2peak_ following ECC training  ↑ incline leg press (6RM) and MVIC following CON and ECC (>↑following ECC training) | 2 |
| Perrey et al.^6^, 2001  *“Comparison of oxygen uptake kinetics…”* | *n=6* men, 25 ± 1 years | CON incremental step test to determine VT and VO_2peak_  3 x 6-min bouts of CON and ECC cycling at low, moderate and heavy intensities, 60 rpm. | ECC cycling work rates were determined based on CON cycling measures.  Low = steady-state VO_2_  Moderate = ~90% VT  Heavy = VT + 0.7Δ | ECC and CON  Low = 62 ± 7 W  Moderate = 207 ± 11 W  Heavy = 317 ± 14 W | 3 (11,12,13) | ECC and CON  Low = 3.7 ± 0.4 %  Moderate = 12.2 ± 0.7 %  Heavy = 18.7 ± 0.8 % | No Δ rate VO_2_ increase for ECC v low CON  ↑ VO_2_ kinetics for ECC and low CON v high CON. | 105 |
| Dufour et al.^7^, 2004  *“Eccentric cycle exercise: training…”* | *n=*8 males, 28 ± 2 years | Participants cycled ECC for 10-min starting at 100 W and progressing to 200 W by final session, 80 rpm. | CON incremental cycle test. ECC cycling intensity determined by peak CON aerobic power output. | ECC = 287 ± 16 W | 1 (14) | ECC = 17 ± 0.9 % | No Δ SV at equivalent VO_2_  ↑ HR 17% during ECC, leading to 27% ↑ Q̇, compared to CON cycling | 44 |
| Dufour et al.^8^, 2007  *“Deciphering the metabolic and mechanical…”* | *n=*11 men, 28±6 years | Experiment = 3 x 6-min of heavy CON (270±13 W), heavy ECC (270±13 W) and light CON (70±9 W) at 60 rpm | Incremental CON cycling step test to volitional exhaustion. 60 rpm, increase of 30 W/min, to determine VO_2max_ and peak PO  ECC cycling performed at a mean intensity of 84% peak PO derived from CON incremental cycling test. | Heavy CON = 270 ± 13 W  Heavy ECC= 270 ± 13 W  Light CON = 70 ± 9 W | 1 (15) | Heavy ECC = 16 ± 0.8 % | ↑ Q̇ (~threefold), ↑ HR (~1.5x) and 13 % ↑ SV for heavy CON v heavy ECC  No Δ VO2 heavy ECC and light CON | 25 |
| Elmer et al.^9^, 2010  *“Joint-specific power absorption…”* | *n=*11 recreationally active males, 24±1 years | Testing:  1. 5-min CON warm up at self-selected intensity  2. 3x maximal inertial load CON cycling test to determined P_max_  3. 60 s semi-recumbent ECC cycling trials @ 20% P_max_ | Maximal CON test to determine CON P_max_  ECC cycling intensity based on 20% of CON P_max_ | ECC = ~ 256 W (single leg = 128 ± 17 W | 1 (16) | ECC = ~15.1 % (single leg = 7.6 ± 1.0 %) | Ankle, knee and hip joints absorbed 10, 58 and 29% of total ECC cycling power.  Main absorption of ECC cycling power occurs within knee extensor muscles (quads),  ECC cycling can be used to preferentially improve knee extensors function and hip flexor muscle cross-sectional area | 18 |
| Elmer, McDaniel & Martin ^10^, 2010  “*Alterations in neuromuscular function…”* | *n=*18 male, recreational cyclists, 31±7 years | Single-leg.  2 x maximal single-leg CON cycling to determine P_max_, test were ~4.5 s on an inertial-load ergometer - upright Monark 81B ergo  Participants performed 5-min single-leg ECC cycling @ 40% of CON P_max_ | Maximal single-leg CON cycling to determine P_max_, test were ~4.5 s. ECC recumbent cycling @ 40% of CON P_max_ | ECC = 151 ± 32 W  CON = 148 ± 21 W | 1 (17) | ECC = 8.9 ± 1.9 % | ↓ CON P_max_ in ipsilateral leg 24 and 48 h post 5-min ECC cycling  ↑ RPE during CON P_max_ testing 24 and 48 h post ECC cycling | 19 |
| Laroche et al.^11^, 2013  *“Is it possible to individualize intensity of eccentric…”* | *n=*11, including three females, 22-37 years | Three cycling bouts.  1. Incremental CON cycle test to an RPE of 12.  2. 20-min steady-state CON cycling  3. 20-min ECC cycling | 20-min of ECC cycling at same CON power achieved at RPE of 12, 15 rpm | ECC = 26.5 ± 9.1 W  CON = 92.0 ± 48.6 W | 1 (18) | ECC = 1.6 ± 0.5 % | CO during ECC v CON cycling  Compared to baseline VO_2_ only a twofold ↑ VO_2_ during ECC cycling compared to fivefold ↑ during CON cycling at equitable power. | 8 |
| Lechauve et al.^12^, 2014  *“Breathing patterns during eccentric exercise…”* | *n=*8 active males, 28±6 years | 2 x incremental cycling tests:  1. CON  2. ECC | CON incremental test, used to terminate incremental ECC test at CON-derived ventilatory threshold | Same VO_2_  CON = 155.0 ± 14.1 W  ECC = 420 ± 30.2 W  Same Power  CON = 262.5 ± 32.0 W  ECC = 262.5 ± 32.0 W | 2 (19,20) | Same VO_2_  ECC = 24.8 ± 1.8 %  Same Power  ECC = 15.5 ± 1.8 % | ↓ (5-fold) VO_2_ and V_E_ during ECC v CON cycling at same power.  ↓ VL EMG during ECC v CON cycling at same power.  ↓ V_t_ during ECC v CON at the same VO_2_  ↑ F_b_ ECC v CON. | 7 |
| Penailillo, Blazevich, Numazawa & Nosaka ^13^, 2013^‡^  *“Metabolic and muscle damage profiles…”* | *n=*10 males, 28.4 ± 8.3 years | 3 x 30-min CON and ECC (ECC1 & ECC2) cycling | Incremental step test on CON recumbent ergometer to determine VO_2peak_/ P_max_  All CON and ECC 30-min cycling at 60% CON P_max_ | CON = 158.5 ± 9.2 W  ECC1 = 169.9 ± 26.7 W  ECC2 = 179.3 ± 6.1 W | 2 (21,22) | ECC1 = 10.0 ± 1.6 %  ECC2 = 10.6 ± 0.4 % | ↓ mean HR, VO_2_, BLa, RPE and EMG lower during ECC1 v CON  ↓ MVC, CMJ and SJ ECC1 v CON  ↑ MS increased ECC1 v CON  ↓ HR and BLa ECC1 v ECC2  No Δ in MVC, CMJ and SJ ECC2 v CON | 37 |
| Penailillo, Blazevich & Nosaka ^14^, 2014^‡^  *“Energy expenditure and substrate oxidation…”* | *n=*10 males, 28.4 ± 8.3 years | 3 x 30-min CON and ECC (ECC1 & ECC2) cycling | Incremental step test on CON recumbent ergometer to determine VO_2peak_/ P_max_  All CON and ECC 30-min cycling at 60% CON P_max_ | CON = 158.5 ± 9.2 W  ECC1 = 169.9 ± 26.7 W  ECC2 = 179.3 ± 6.1 W |  | ECC1 = 10.0 ± 1.6 %  ECC2 = 10.6 ± 0.4 % | ↓ Energy expenditure and carbohydrate for ECC1 and ECC2 v CON.  ↑ fat use during ECC1 (72%) and ECC2 (85%), v CON, and 48% greater during ECC2 v ECC1. | 14 |
| Penailillo et al.^15^, 2015a  *“Rate of force development…”* | *n=*10 males, 28.4±8.3 years | 3 x 30-min cycling tasks at 60 rpm:  1. CON  2. ECC1  3. ECC2 | ECC cycling was performed for 30-min at 60% CON P_max_ derived from CON incremental step test | CON = 158.5 ± 9.2 W  ECC1 = 169.9 ± 26.7 W  ECC2 = 179.3 ± 6.1 W |  | ECC1 = 10.0 ± 1.6 %  ECC2 = 10.6 ± 0.4 % | ↓ MVIC peak torque for ECC1 v CON and ECC2 | 40 |
| Penailillo, Blazevich & Nosaka ^16^, 2015b  *“Muscle fascicle behaviour during eccentric…”* | *n=*11 males, 27.1±7.0 years | 2 x 10-min ECC cycling @ 65% CON P_max_ | All 10-min CON and ECC cycling completed at 65% P_max_ derived from CON incremental step test | ECC1 = 189.7 ± 43.2 W  ECC2 = 194.0 ± 48.5 W | 2 (23,24) | ECC1 = 11.2 ± 2.6 %  ECC2 = 11.5 ± 2.9 % | ↑ MS ECC1 v ECC2  No Δ MVIC, EMG, peak torque  ↓ 16% Fascicle elongation for ECC2, v ECC1. | 23 |
| Penailillo, Blazevich & Nosaka ^17^, 2017  *“Factors contributing to lower metabolic demand…”* | *n=*11 untrained, but active men, 27.1±7.0 years | 2 x 10-min CON and ECC cycling @ 65% CON P_max_ | All 10-min CON and ECC cycling completed at 65% P_max_ derived from CON incremental step test | ECC = 194.0 ± 48.5 W  CON = 188.6 ± 40.8 W | 2 (25) | ECC = 11.5 ± 2.9 % | ↓ mean VO_2_ and HR for ECC v CON  total oxidation index greater for ECC v CON  ↓ VL, VM, RF and BF peak EMG amplitude during ECC v CON. | 7 |
| Penailillo et al.^18^, 2017  *“Metabolic demand and muscle damage induced…”* | *n=*8 males, 23.3±0.7 years | 2 x 30-min ECC cycling tasks at 60 rpm:  1. EXT  2. FLEX | ECC cycling was performed for 30-min at 60% CON P_max_ derived from CON incremental step test | ECC EXT = 168.2 ± 25.8 W  ECC FLEX = 150.6 ± 32.2 W | 2 (26,27) | ECC EXT = 9.9 ± 1.5 %  ECC FLEX = 8.9 ± 1.9 % | ↑ VO_2_, HR, RPE FLEX v EXT  ↓ CMJ and SJ for FLEX and EXT  ↑ MS EXT v FLEX | 3 |
| Penailillo, Mackay & Abbiss ^19^, 2018a  *“Rating of perceived exertion during concentric and eccentric…”* | *n=*10 males, 29.8±2.3 years | Two visits:  1. CON incremental step test  2.4x5-min bouts ECC cycling or CON cycling | CON and ECC cycling visits performed at 30, 60, 80 and 100% CON P_max_, derived from step test | ECC  @ 30% = ~100 W  @ 60% = ~200 W  @ 90% = ~250 W  @ 100% = ~450 W | 4 (28,29,30,31) | ECC  @ 30% = ~5.9 %  @ 60% = ~11.8 %  @ 90% = ~ 14.8 %  @ 100% = ~26.6 % | ↑ RPE at 30, 60, 80 and 100% for CON v ECC  No Δ PE CON v ECC  ↑ HR and VO_2_ CON v ECC | 5 |
| Penailillo et al.^20^, 2018b  *“Effect of eccentric cycling performed at…”* | *n=*20 males, 20-27 years separated in to two groups:  1. LONG (*n*=10)  2. SHORT (*n*=10) | Two visits:  1. CON incremental step test  2.30-min ECC cycling at LONG or SHORT muscle length | ECC cycling was performed for 30-min at 80% CON P_max_ derived from CON incremental step test | ECC LONG = 191.8 ± 23.6 W  ECC SHORT = 211.8 ± 23.4 W | 2 (32,33) | ECC LONG = 11.3 ± 1.4 %  ECC SHORT = 12.5 ± 1.4 % | ↑ HR and RPE LONG v SHORT  ↓ MVIC LONG v SHORT  ↓ VM pressure-pain threshold LONG v SHORT | 2 |
| Rakobowchuk et al.^21^, 2018  *“Muscle Oxygenation Responses…”* | *n=*12 males, 29.2±6.6 years | 2 x 45-min CON and ECC cycling at CON, 30 rpm | CON incremental cycling test to determine VO_2peak_ and peak HR  ECC cycling intensity set at 54% peak HR | CON = 82 ± 16 W  ECC = 210 ± 40 W | 1 (34) | ECC = 12.4 ± 2.4 % | ↑ (2.5x )power output ECC v CON  No Δ VO_2_, BLa, CO and systolic arterial pressure ECC v CON  ↑ Diastolic and mean blood pressures during ECC v CON  No Δ muscle O_2_ profiles | 3 |

‡ denotes that Penailillo et al. 2013, 2014 and 2015a used the same testing protocol and reported the same ECC cycling power output values.

Abbreviations: BF Biceps femoris**;** Q̇ Blood flow; F_b_ Breathing frequency; BLa Blood lactate; CO Cardiac output; Δ change in; CON Concentric; CMJ Countermovement jump; ↓ Decrease(s); ECC Eccentric; V_E_ expired volume; EXT Extension; FLEX Flexion; ↑ Increase(s); HR Heart rate; ISO Isometric; P_max_ Maximal CON cycling power output; MVIC Maximal voluntary isometric contraction; MS Muscle soreness; VO_2peak_ Peak oxygen consumption; PE Perceived exertion; MHR Percentage of age-predicted maximal heart rate; PO Power output; VO_2/max_ Oxygen consumption; RF Rectus femoris; SJ Squat jump; SV Stroke volume; V_t_ Tidal volume; VM/L Vastus medialis/lateralis; VT Ventilatory threshold.

**References**

1. Gross M, Lüthy F, Kroell J, Müller E, Hoppeler H, Vogt M. Effects of eccentric cycle ergometry in alpine skiers. *Int J Sports Med.* 2010; 31(08):572-576.

2. Vogt M, Hoppeler H. Competitive Alpine Skiing: Combining Strength and Endurance Training: Molecular Bases and Applications. *Science and Skiing V.* 2012; 5.

3. Elmer S, Hahn S, McAllister P, Leong C, Martin J. Improvements in multi‐joint leg function following chronic eccentric exercise. *Scand J Med Sci Spor.* 2012; 22(5):653-661.

4. Leong C, McDermott W, Elmer S, Martin J. Chronic eccentric cycling improves quadriceps muscle structure and maximum cycling power. *Int J Sports Med.* 2014; 35(07):559-565.

5. Lewis MC, Peoples GE, Groeller H, Brown MA. Eccentric cycling emphasising a low cardiopulmonary demand increases leg strength equivalent to workload matched concentric cycling in middle age sedentary males. *J Sci Med Sport.* 2018; 21(12):1238-1243.

6. Perrey S, Betik A, Candau R, Rouillon JD, Hughson RL. Comparison of oxygen uptake kinetics during concentric and eccentric cycle exercise. *J Appl Physiol.* 2001; 91(5):2135-2142.

7. Dufour SP, Lampert E, Doutreleau S, et al. Eccentric cycle exercise: training application of specific circulatory adjustments. *Med Sci Sports Exerc.* 2004; 36(11):1900-1906.

8. Dufour SP, Doutreleau S, Lonsdorfer-Wolf E, et al. Deciphering the metabolic and mechanical contributions to the exercise-induced circulatory response: insights from eccentric cycling. *Am J Physiol Regul Integr Comp Physiol.* 2007; 292(4):1641-1648.

9. Elmer SJ, Madigan ML, LaStayo PC, Martin JC. Joint-specific power absorption during eccentric cycling. *Clin Biomech.* 2010; 25(2):154-158.

10. Elmer SJ, McDaniel J, Martin JC. Alterations in neuromuscular function and perceptual responses following acute eccentric cycling exercise. *Eur J Appl Physiol.* 2010; 110(6):1225-1233.

11. Laroche D, Joussain C, Espagnac C, et al. Is it possible to individualize intensity of eccentric cycling exercise from perceived exertion on concentric test? *Arch Phys Med Rehab.* 2013; 94(8):1621-162.

12. Lechauve J, Perrault H, Aguilaniu B, et al. Breathing patterns during eccentric exercise. *Resp Physiol Neurobi.* 2014; 202:53-58.

13. Peñailillo L, Blazevich A, Numazawa H, Nosaka K. Metabolic and muscle damage profiles of concentric versus repeated eccentric cycling. *Med Sci Sports Exerc.* 2013; 45(9):1773-1781.

14. Peñailillo L, Blazevich A, Nosaka K. Energy expenditure and substrate oxidation during and after eccentric cycling. *Eur J Appl Physiol.* 2014; 114(4):805-814.

15. Peñailillo L, Blazevich A, Numazawa H, Nosaka K. Rate of force development as a measure of muscle damage. *Scand J Med Sci Spor.* 2015; 25(3):417-427.

16. Peñailillo L, Blazevich AJ, Nosaka K. Muscle fascicle behavior during eccentric cycling and its relation to muscle soreness. *Med Sci Sports Exerc.* 2015; 47(4):708-717.

17. Penailillo L, Blazevich AJ, Nosaka K. Factors contributing to lower metabolic demand of eccentric than concentric cycling. *J Appl Physiol.* 2017; 123(4):884-893.

18. Peñailillo L, Guzmán N, Cangas J, Reyes A, Zbinden-Foncea H. Metabolic demand and muscle damage induced by eccentric cycling of knee extensor and flexor muscles. *Eur J Sport Sci.* 2017; 17(2):179-187.

19. Peñailillo L, Mackay K, Abbiss CR. Rating of perceived exertion during concentric and eccentric cycling: Are we measuring effort or exertion? *Int J Sport Physiol Perform.* 2018; 13(4):517-523.

20. Peñailillo L, Aedo C, Cartagena M, et al. Effects of Eccentric Cycling Performed at Long vs. Short Muscle Lengths on Heart Rate, Rate Perceived Effort, and Muscle Damage Markers. *J Strength Cond Res.* 2018.

21. Rakobowchuk M, Isacco L, Ritter O, et al. Muscle Oxygenation Responses to Low-intensity Steady Rate Concentric and Eccentric Cycling. *Int J Sports Med.* 2018; 39(3):173-180.
